# Interannual Variability of Cyanobacterial Blooms in Lake Erie

**DOI:** 10.1371/journal.pone.0042444

**Published:** 2012-08-01

**Authors:** Richard P. Stumpf, Timothy T. Wynne, David B. Baker, Gary L. Fahnenstiel

**Affiliations:** 1 National Oceanic and Atmospheric Administration (NOAA), National Centers for Coastal Ocean Science, Silver Spring, Maryland, United States of America; 2 Heidelberg University, National Center for Water Quality Research, Tiffin, Ohio, United States of America; 3 NOAA, Great Lakes Environmental Research Laboratory, Muskegon, Michigan, United States of America; University of Vigo, Spain

## Abstract

After a 20-year absence, severe cyanobacterial blooms have returned to Lake Erie in the last decade, in spite of negligible change in the annual load of total phosphorus (TP). Medium-spectral Resolution Imaging Spectrometer (MERIS) imagery was used to quantify intensity of the cyanobacterial bloom for each year from 2002 to 2011. The blooms peaked in August or later, yet correlate to discharge (Q) and TP loads only for March through June. The influence of the spring TP load appears to have started in the late 1990 s, after Dreissenid mussels colonized the lake, as hindcasts prior to 1998 are inconsistent with the observed blooms. The total spring Q or TP load appears sufficient to predict bloom magnitude, permitting a seasonal forecast prior to the start of the bloom.

## Introduction

**Figure 1:**
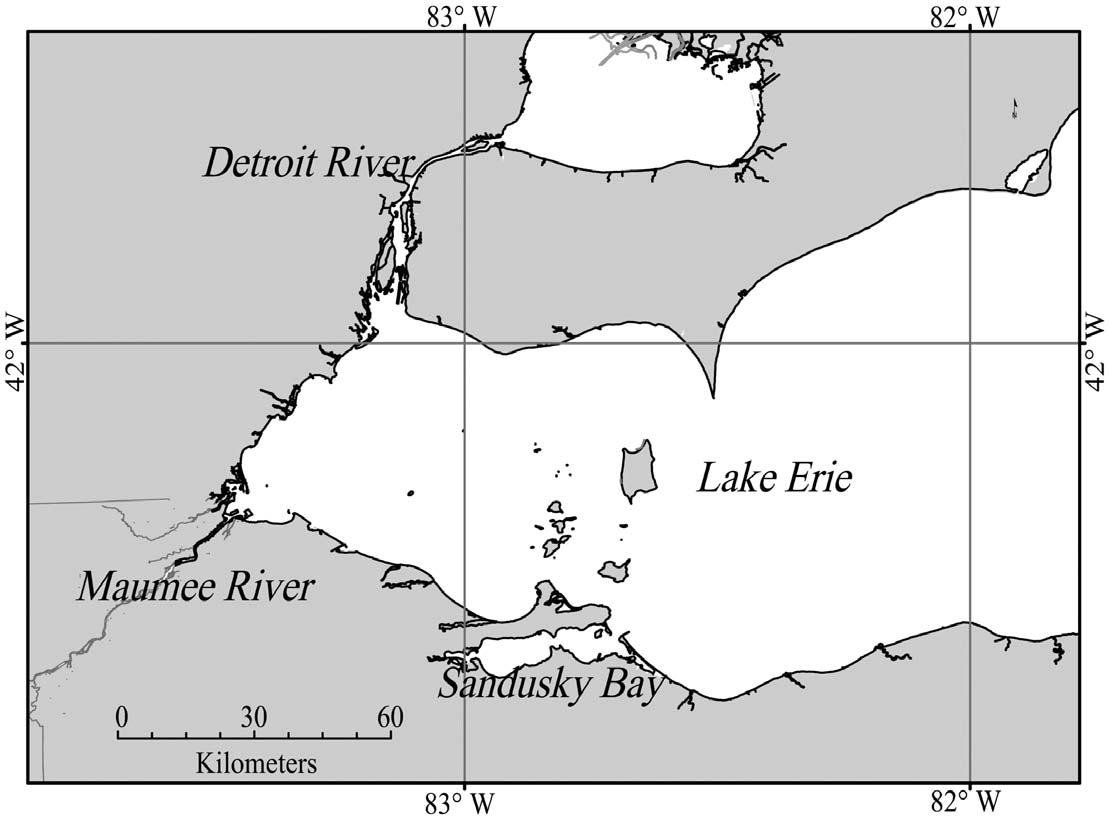
Map of western Lake Erie.

Lake Erie suffered from intense blooms of cyanobacteria in the 1970 s. Following phosphorus abatement strategies these blooms disappeared in the 1980 s [1]–[3]. The blooms reappeared in the 1990 s, with blooms dominated by *Microcystis aeruginosa* common in the last decade [4]–[5]. During this time the annual total phosphorus (TP) load has not changed, but annual soluble reactive phosphorus (SRP) loads have increased [6]. In addition, the 1990 s saw ecological disruptions caused by invasive mussels of the genus *Dreissena*, which have been hypothesized to promote cyanobacterial blooms [7]–[9]. The Maumee River (Figure 1), the single largest watershed draining into the Laurentian Great Lakes, has also been hypothesized to supply the needed nutrients to fuel the *Microcystis* spp. blooms [5]. Over 80% of the land within the watershed is used for agriculture [10], and it discharges into the shallowest portion of Lake Erie.

*Microcystis* produce noxious and toxic compounds that cause a variety of detrimental impacts [11]. These impacts include animal mortalities and human health risks from the toxin microcystin, as well as taste and odor problems in finished drinking water, if not specifically treated [11]–[12]. Therefore a seasonal prediction of the blooms would aid managers in planning mitigation strategies. Warmer temperatures may exacerbate blooms, increasing the severity of these impacts [13].

Satellite imagery can provide data on the areal extent of cyanobacterial blooms [14]–[16]. Of the several instruments, the Medium-spectral resolution imaging spectrometer (MERIS) permits quantification of blooms even in water with suspended sediments [17]–[18], including Lake Erie [15], [19]. MERIS data is available since 2002, allowing a comparison of the bloom intensity with Maumee River loads for each of the last ten years. With several bands in the red and the “red edge” portion of the near-infrared, MERIS data allow spectral shape algorithms that can target severe blooms [19]–[21]. Spectral shape methods use a computational equivalent to the second derivative [15]; these include fluorescent line height (FLH) [20]; maximum chlorophyll index (MCI) [21], and the cyanobacteria index (CI) [19]. The MCI was demonstrated to be effective in coastal ocean blooms with data that has not been atmospherically corrected [21]. This power of spectral shape algorithms means that far more data can be retrieved under thin cloud and glint conditions than with standard algorithms based on water-leaving radiance. The CI, which is the negative of the FLH [20], has been quite effective at identifying cyanobacterial blooms in Lake Erie [15], [19], and appears to be less sensitive to high sediment loads than the MCI.

## Methods

### Satellite

MERIS reduced resolution (nominal 1.1 km width) daily imagery was acquired from the European Space Agency (ESA) as standard level 2 water reflectance created by the second reprocessing [22]. The imagery was mapped to a 1.1 km Sinusoidal equal area projection using nearest neighbor sampling. The Cyanobacterial Index (CI) was calculated as described by Wynne et al. [15], [19] using the spectral shape around 681 nm band:


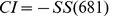
(1)

The spectral shape (*SS*), or curvature is determined as a nominal second derivative around the band of interest:


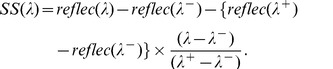
(2)

where *λ* = 681 nm (MERIS band 8), *λ* ^+^ = 709 nm (band 9), and *λ* ^−^ = 665 nm (band 7). An equivalent computation with these bands also produces the FLH [20]. L2 reflectances are used for the analysis, these are normalized water-leaving reflectances for pixels identified as water. The method could be applied to the L1 (radiance) products, but use of L2 allows use of the ancillary products, like the cloud mask, as well as simplifying comparisons to other sensors or field instruments.

Using the individual CI images, 10-day composites were calculated by taking the highest CI at each pixel available from any of the daily images within a given 10-day time period to remove clouds and capture the areal biomass; the latter because *Microcystis* will aggregate at the surface providing effective detection with remote sensing [15], [18], [19]. Under clear water conditions, the bands used for the CI do not detect light from deeper than one meter [15]; adding particulate matter will increase light attenuation, further reducing depth penetration. Images that showed artifacts from atmospheric correction failure were not used for analysis (6 daily images from over 400 used in the entire time series).

**Figure 2:**
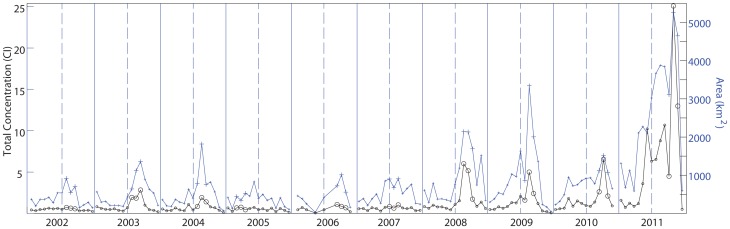
Time series of bloom intensity (black) and area >0.001 CI (blue) from the cumulative CI for each 10-day composites. The intensity is the sum of CI-values at all pixels within the image. The area is determined from the total number of pixels with CI >0.001. Circles mark the three composites used to determine the annual severity. Each year has 15 composites, from June 1–10 to October 19–28. The dashed grid line marks the August 10–19 period.

**Table 1:** Annual CI, and bloom area (>0.001 CI equivalent to >10^5^ cells mL^−1^) for each year, the average Q for March to June, and the total TP and SRP loads for March to June, and total TP load for June only.

year	CI	Bloom area km^2^	Q average Mar-Jun m^3^ s^−1^	TP total Mar-Jun m.tons	TP total Jun m.tons	SRP totalMar-Jun m.tons	Ratio TP_June_/TP_March-May_	CI predicted from exponential Q	CI predicted from TP for Jun
1995		Bloom reported	200	867	73	68	0.09	0.58	1.68
1996			311	1303	304	144	0.30	3.05	5.68*
1997			310	1303	445	187	0.52	3.02	8.10*
1998		Bloom reported	262	1306	161	171	0.14	1.61	3.19
1999			235	803	61	105	0.08	1.06	1.47
2000			226	941	396	145	0.73	0.92	7.26*
2001			181	489	93	107	0.24	0.40	2.02*
2002	0.64	170	262	870	27	150	0.03	1.60	0.87
2003	2.20	705	297	1409	121	322	0.09	2.55	2.50
2004	1.38	451	255	892	508	196	1.32	1.44	9.20*
2005	0.59	198	119	309	14	84	0.05	0.08	0.66
2006	0.87	293	175	577	58	124	0.11	0.35	1.41
2007	0.83	288	222	1048	13	258	0.01	0.86	0.64
2008	4.30	1047	318	1315	219	265	0.20	3.32	4.20
2009	2.86	992	310	1327	88	203	0.07	3.02	1.93
2010	3.75	1050	330	1269	205	312	0.19	3.82	3.97
2011	14.18	2968	473	2240	102	419	0.05	14.9	2.18

* indicates TP_June_/TP_March-May_ ratio of >0.2.

Creating the composites served two purposes. The first is to establish an image set that is virtually free of clouds, thereby negating the need to account for missing (cloud-obscured) pixels. The second is to best approximate the areal biomass through the season. *Microcystis* is typically positively buoyant, especially during morning (when MERIS collects data), such that it accumulates at the surface during calm conditions [15], [18], although mixes through the water column during substantial winds (>7.7 m s^−1^ in Lake Erie) [15]. The CI is an estimate of surface concentration, which includes all the biomass during calm winds, but underestimates the bloom biomass under high winds. In western Lake Erie, 2–4 days of calm weather are common each week in the summer, allowing the cells to concentrate at the surface [15]. The maximum CI during the 10-day period provides a measure of the total biomass at each pixel.

**Figure 3:**
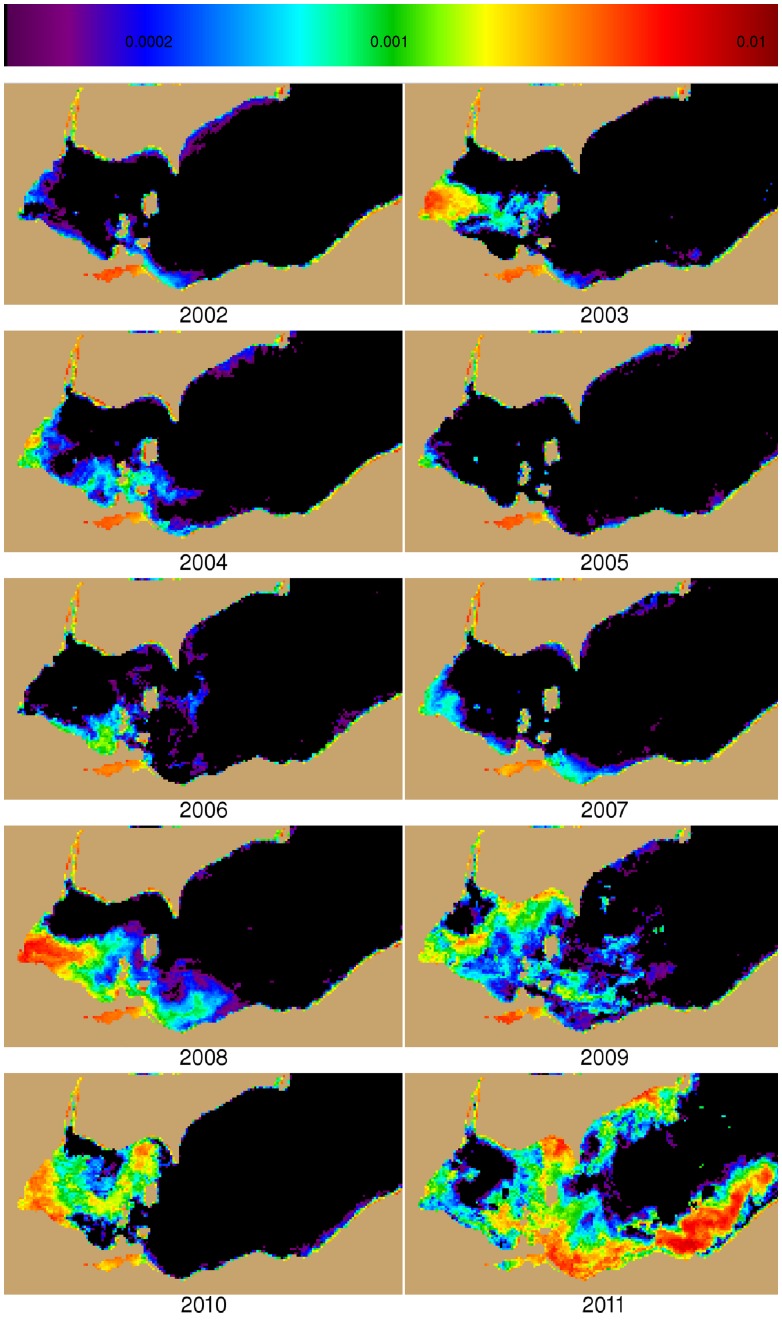
Mean of the three 10-day composites (identified by circles in **Figure 2**) used to compute intensities for each year for western Lake. Black indicates CI< = 0. Brown is land. Maumee River input is the far western end of the lake (see Figure 1).

We calculated intensity (biomass), for each of the 10-day composite images. The intensity was derived by summing the value of the CI at all pixels within the potential bloom area of the each composite. Only a few composites had missing data because of clouds, but these did not coincide with peak bloom concentrations. The data from 2006 was particularly gappy with some 10-day composites having no available imagery. However, the gaps did not impact the analysis owing to the early timing of the missing images and the relatively low cyanobacterial biomass that was present that year. While cyanobacteria may dominate for a few months, the intense bloom typically lasted 30–40 days (Figure 2). The annual bloom severity (Table 1) was determined by averaging the highest three consecutive 10 day composites (Figure 3). From July to October, cyanobacteria overwhelmingly dominated the biomass in areas with measurable CI [4], [5], [15], [19]. We also determined the bloom area by counting the total number of pixels which had CI >0.001, nominally equivalent to bloom concentration of 10^5^ cells mL^−1^ [15], the threshold for severe blooms [23]. For the annual peak blooms, the average density was 301 km^2^ CI^−1^ with a standard deviation of 48 km^2^ CI^−1^.

**Figure 4:**
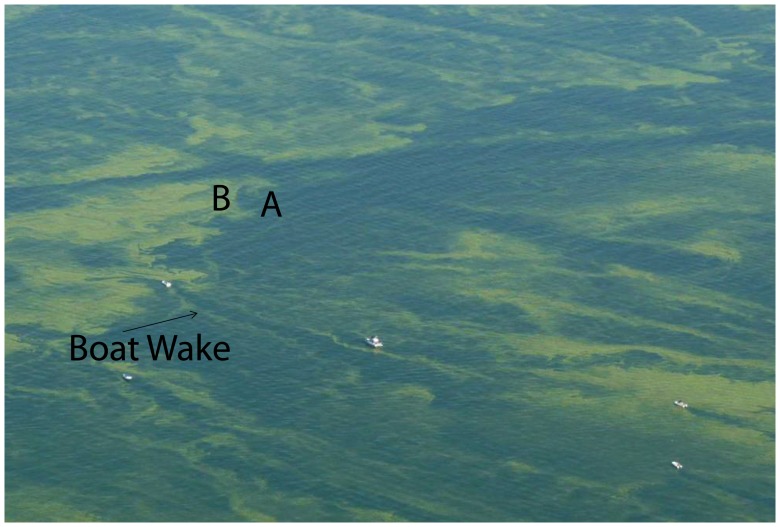
Aerial photograph of *Microcystis* bloom in western Lake Erie, early in the 2009 bloom, showing the spatial variability during intense blooms. “A” shows less dense and mixed bloom, “B” shows denser bloom aggregating at the water surface. Mixing can be seeing within the boat wake. Image width is approximately 400 m (credit Tom Archer).

The intensity estimate is a more robust statistic than the area because it gives the total biomass of the bloom. For this reason all statistical analysis will be done with intensities; however Figure 2 and Table 1 show that the area and biomass exhibit similar trends, so the area relationships can be inferred with the density model.

**Table 2:** R^2^ for all 10 years between maximum CI and the load of interest for months with positive correlation.

Time	Q r^2^	TP r^2^	SRP r^2^
February	0.003	0.033	0.023
March	0.401	0.319	0.329
April	0.283	0.336	0.094
May	0.550	0.519	0.481
June	0.011	0.0011	0.003

March-May and March-June are the average of the months for Q and the cumulative for TP and SRP.

p-values: r^2^>0.3, p<0.10; r^2^>0.4, p<0.05.

Blooms in Lake Erie in summer are predominately *Microcystis* [4], [5], [15]. *Planktothrix* is common in Sandusky Bay, but has been found in significant concentrations in the western basin only in 2005, a non-bloom year [4]. (From 2008 to 2011, field sampling as part of the Lake Erie Harmful Algal Bloom Bulletin, identified *Microcystis* as the bloom-forming organism [24].) Sandusky Bay was not included in the analyses, as its blooms are locally driven and typically remain within the Bay. The relationship between CI and cell concentration reported in [15] is based on *Microcysti*s. The r^2^ of 0.42, while highly significant statistically (p<0.001) is probably reduced by the comparison of different spatial scales: pixels against water samples in these highly patchy blooms (Figure 4). Cyanobacteria blooms often have concentrations varying orders of magnitude in the space of a 1-km pixel [25]. However, in the context of this study, the relationship of CI to concentration [15] is sufficiently accurate, although with a potential to underestimate as much as a factor of 2. As more data is collected in the future, the relationship should be better constrained. Our use of CI in this study, rather than cell concentration, will assure that any future adjustments in the relationship will not significantly alter these results.

**Table 3:** R^2^ and residual standard error (RSE) for all 10 years and bloom years between maximum CI and the combined loads of March to May, March to June (in bold), and March to July.

Time	Q r^2^	Q RSE (as CI)	TP r^2^	TP RSE (as CI)	SRP r^2^	SRP RSE (as CI)
Mar-May all years	0.75	2.2	0.71	2.3	0.57	2.9
**Mar-June all years**	**0.74**	**2.2**	**0.75**	**2.2**	**0.60**	**2.8**
Mar-July all years	0.64	2.6	0.63	2.7	0.40	3.4
Feb-June all years	0.68	2.4	0.64	2.6	0.54	2.9
Mar-May bloom years	0.84	2.1	0.75	2.7	0.61	3.3
**Mar-June bloom years**	**0.97**	**0.96**	**0.89**	**1.7**	**0.65**	**3.1**
Mar-July bloom years	0.58	3.4	0.63	2.7	0.26	4.5
Feb-June bloom years	0.69	2.9	0.56	3.5	0.43	4.0

P-value thresholds, for all years: r^2^>0.4, p<0.05; r^2^>0.58, p<0.01; r^2^>0.74, p<0.0014.

P-value thresholds for bloom years: r^2^>0.66, p<0.05; r^2^>0.84, p<0.01; r^2^>0.84, p<0.01.

June has a strong influence on the statistics for the bloom years.

### Water Temperature

There are no buoys that have routinely collected temperature data in the western basin over the ten years. Water temperature was obtained from monthly mean 4-km data sets of the standard 11-micron night-time sea surface temperature algorithm of the Moderate Resolution Imaging Spectro-radiometer (MODIS) on the AQUA satellite. Averages for each month for the western basin (west of 82.5W) were obtained from NASA’s Giovanni web site with an accuracy of +/−0.4°C [26]. The temperature data shows that July and August are the hottest months, and the temperature does not rise above 20°C until June. This may partly explain the lag in development of the blooms, the water temperatures until June are not optimal for *Microcystis* growth.

**Figure 5:**
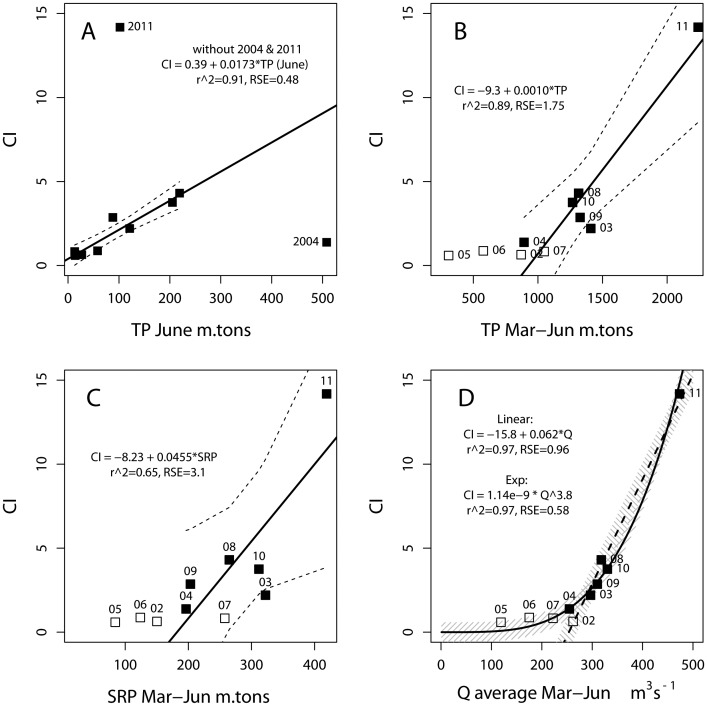
Bloom intensity from 2002 to 2011 compared to loads with CI of 1 equivalent to 300 km^2^ of 10^5^ cells mL^−1^ (A) TP for June, with regression for eight years excluding 2004 and 2011; (B) cumulative TP for March to June with regression (and regression confidence interval) for bloom years (filled squares); (C) cumulative SRP for March to June like (B) with regression line and confidence for bloom years; (D) average Q for March to June, with linear and exponential regression for bloom years. Shading in (d) indicates the RSE for each of the two regressions, which closely matches the regression confidence (except near 2011).

### River Discharge

River discharge (Q) was obtained from the United States Geologic Survey (USGS) Waterville, Ohio Station on the Maumee River (Station 04193500) [27]. Monthly average discharge was calculated from the daily averages. For water years (October 1 of the previous year to September 30) of 2010 and 2011, the monthlies were determined from USGS provisional data, as official data was not yet available.

**Figure 6:**
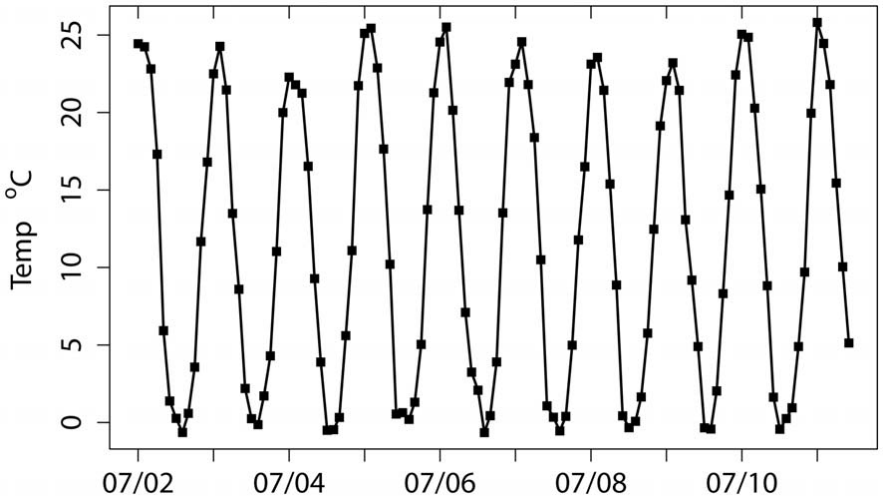
Monthly mean water temperature in western Lake Erie from 2002–2011. Tick marks indicate July of each year.

### Phosphorus and Nitrogen Loads

A refrigerated autosampler is used to collect samples near the Waterville United States Geological Survey (USGS) Stream Gage. Samples are returned to Heidelberg University’s National Center for Water Quality Research (NCWQR) at weekly intervals, where three samples per day are analyzed during high flow periods and one per day during low flows. Total phosphorus (TP) and soluble reactive phosphorus (SRP) are analyzed using EPA Method 365.1 and nitrogen as nitrate + nitrite (NOx) is analyzed using EPA Method 300.1 [28], [29]. Nitrite is negligible compared to nitrate [28]. Additional details of the sample collection and analytical methods are presented at the tributary loading section of the NCWQR’s website [28].

Monthly loads (m.tons) were calculated from daily loads determined from flow-weighted concentration [30]. Table 1 gives the totals for spring (March to June) used in this paper. CI was compared with Q, TP, and SRP using standard least squares regression, including determination of p-values and residual standard error (RSE) to determine significance of the models [31].

**Figure 7:**
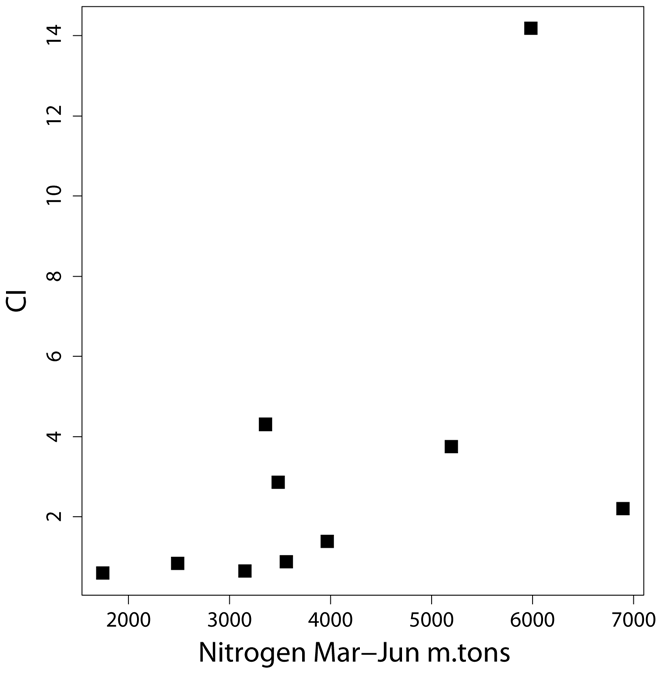
Spring (March to June) load of NOx nitrogen compared to CI showing the lack of a relationship (r^2^ = 0.29, p = 0.11).

### Bloom Phosphorus Calculation

For comparison with TP values, the phosphorus load within a bloom was calculated based on literature values for phosphorus in cells and potential cells in the bloom. Phosphorus within the *Microcystis* cells in Lake Erie has been determined from measured intra-cellular phosphorus per dry weight [32]. The calculation used phosphorus per cell (0.26 mmoles P per kg dry wt [32]), which gives 8.1 mg P per g dry wt. The literature values for dry weight per cell are 17–43 pg [33]. The resulting phosphorus (given the range in dry weight per cell) is 143 to 352 fg P cell^−1^. We now need to equate the intra-cellular phosphorus to the phosphorus in the bloom observed from satellite. A CI of 0.001 corresponds to a concentration of about 10^5^ cells mL^−1^ [15], which has phosphorus concentration of 14.3 to 35.2 ng P mL^−1^. If we assume the bloom is concentrated within 1 meter of the surface (because of buoyancy), there are 1.2 *10^9^ L within the pixel of area 1.21 km^2^. This results in 17 to 42 kg intra-cellular P in the bloom for a CI of 0.001 (with a range varying based on the dry weight per cell). An accumulated CI of 1.0 would hold 17 to 42 m.tons P. Current uncertainty in the conversion of CI to cell concentration of about two-fold means that a bloom of CI = 1.0 could contain up to 84 m.tons P. A bloom concentrated closer to the surface would reduce the estimated phosphorus load in the bloom, but does not impact the subsequent conclusions (covered in the Discussion).

**Table 4:** R^2^ and residual standard error (RSE), excluding 2011 from the analysis. Q exp is exponential relationship, Q lin is linear relationship.

Time	Q lin r^2^	Q exp. RSE(as CI)	Q lin r^2^	Q lin RSE(as CI)	TP r^2^	TP RSE(as CI)	SRP r^2^	SRP RSE(as CI)
Mar-Jun 2002–2010	0.62	0.87	0.64	0.91	0.57	1.0	0.44	1.1

P-value thresholds: r^2^>0.45, p<0.05; r^2^>0.63, p<0.01.

## Results

The later years, 2008–2011, all show strong blooms (Figures 2, 3); 2003 and 2004 were also considered to be bloom years owing to relatively high intensity (Table 1, Figure 2). Peak blooms occurred in August or September in all years except 2011, which peaked in early October. Only March, April, and May had statistically significant correlations with the annual CI concentration (Table 2). CI is correlated with Q and TP for March, April (at p = 0.06), and May; but only for March and May for SRP. These correlations indicate that spring loads should be examined more closely.

The role of the spring months is more evident in a linear model of the cumulative load for sequential months. For March to May, CI against the TP had an r^2^ = 0.71 and RSE  = 2.32 CI, and Q had an r^2^ = 0.75, with RSE  = 2.17 CI. When additional months were added to the March to May average, poorer correlations occurred, except for the addition of June (Table 3). In particular, when examining the relationships for only the bloom years, the total TP or Q for March to June explained 89% and 97% of the variance, respectively.

June had a unique pattern between loads and CI. While CI was not correlated with June loads for all 10 years, if 2004 and 2011 are excluded, CI is highly correlated against TP (r^2^ = 0.91, RSE  = 0.48 CI, Figure 5A), Q (r^2^ = 0.86, RSE  = 0.61 CI), and SRP (r^2^ = 0.82, RSE  = 0.69 CI). If 2004 and 2011 are excluded from the regression for any other month other than June, the correlation for that month (that is CI to Q, SRP, or TP) becomes lower (r^2^<0.1) or negative. The two excluded years have extreme differences in the contribution of June loads to the cumulative spring (March to June) load. While 2004 had a high TP load in June (Table 1, Figure 5a), it had the 2^nd^ lowest TP load for March-May (after the non-bloom year of 2005), and a relatively low CI. 2011 had a moderate June TP load, but it saw the highest TP load for March to May–70% greater than the March to May load for the next highest year– and the highest observed CI of the time series. Therefore, the spring months are needed to fully explain bloom severity and variability.

Is the spring load of TP sufficient to support peak blooms? As described in the methods, an accumulated CI of 1 corresponds to 17–42 m.tons of intra-cellular P. The 2008–2010 blooms would have contained between 60 and 127 m.tons of P, up to 10% of the average spring TP load and less than the June TP load (Table 1). This indicates that even with losses of TP to the sediments, the Maumee River provides sufficient P to support the blooms.

**Figure 8:**
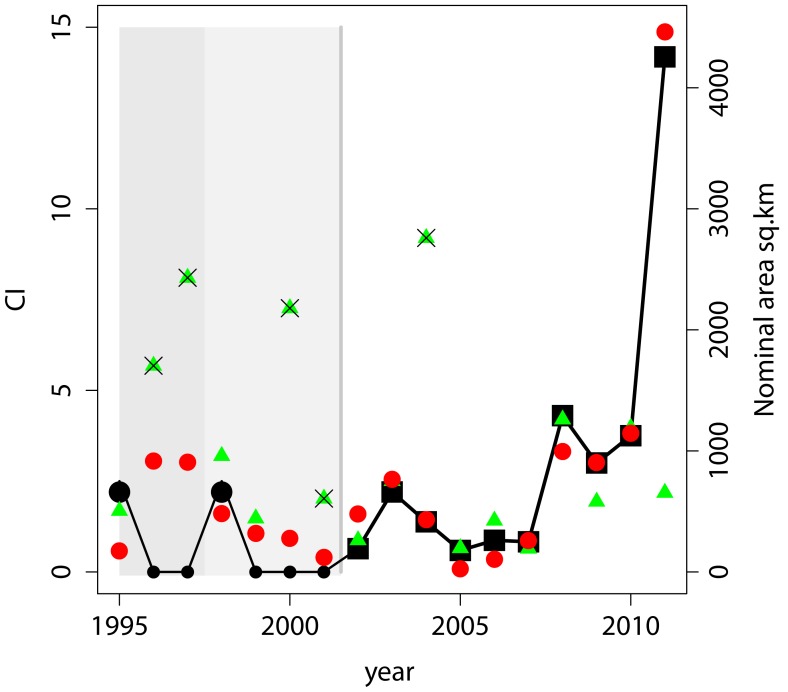
Observed and modeled CI. CI determined here (black squares) from 2002–2011. Period of projected CI is shown in light gray, with black circles giving projected CI based on reports of blooms or not. 1995 and 1998 blooms are assumed to be equal to 2003, other years are assumed zero (no bloom) based on lack of reports. Red circles are CI estimated from exponential Q model. Green triangles are CI from TP for June. Triangles with an X have ratio of TP_June_/TP_March-May_ >0.2. Dark gray shade marks time period when the models fail to predict occurrence or absence of blooms.

### Other Environmental Factors

Cyanobacterial blooms favor warm temperatures [13]. June is the first month when the temperature exceeds 20°C, and the warmest water occurs consistently from July to September (Figure 6). This means that the “lag” months between load and bloom are the warmest in the lake, certainly favoring development of *Microcystis* blooms. Interannual differences in summer temperatures, however, do not explain the variations in bloom intensity within this data set. Warm summers occurred in both non-bloom (2005–2006) and bloom (2010–2011) years. Confirming the lack of relationship, the r^2^ of CI to the average summer temperature (July to September) or any month from May to August, had r^2^ of <0.1 (p values >0.4).

Nitrogen (NOx) load does not show a significant influence on bloom intensity. March to June total load of NOx has r^2^ = 0.29 (p-value of 0.11) and RSE  = 3.7 CI, which is much poorer than the phosphorus relationship (Figure 7). It also appears that ample nitrogen enters Lake Erie in the spring, an average of 15,930 m.tons as nitrate from 2002–2011. With blooms holding about 100 m.tons of P, 20-fold more nitrogen is provided than the 723 m.tons N that would be needed assuming the Redfield ratio of 16∶1 (molar).

### Model of Bloom Intensity

A predictor of bloom intensity should concentrate on the bloom years. For the six years with major blooms (Figures 5b, c, d), spring Q produced a stronger relationship to bloom intensity than TP or SRP (Figures 5b, c, d). Spring TP shows a strong correlation (r^2^ = 0.89; p<0.001), but a large uncertainty (RSE of 1.8 CI). In contrast, CI against the average spring Q has an RSE of 0.96 CI (1/2 of the error for TP) with an improved r^2^ = 0.97. Fitting an exponential model of CI against Q (log CI vs. log Q) delivers than same r^2^ = 0.97 (p = 0.0004) but an improved RSE of 0.58 CI. A CI of 0.58 is equivalent to 12% of the average intensity of the bloom years. (For reference a linear model using March to May for the bloom years has much greater error: for TP, March to May has r^2^ = 0.75 and RSE  = 2.7 CI; and Q has r^2^ = 0.84 and RSE  = 2.1 CI).

### 2011 Bloom

The bloom in 2011 was so severe that it may appear as an outlier in the data set. To determine whether it would skew the results, we examined the data excluding 2011. As noted previously, TP for June can explain the annual peak CI if 2004 and 2011are excluded (because of the extreme difference in loads of March to May). The relationships of Q and TP against CI without 2001 have r^2^>0.57 (Table 4) and are statistically significant at p<0.01 (while SRP has a r^2^ = 0.44 and p = 0.051). We further used exponential and linear models of Q against CI determined from 2002–2010 bloom years to predict the 2011 event. These models were robust; the exponential model had r^2^ = 0.90 (p = 0.013) and RSE  = 0.54 CI, and the linear model had r^2^ = 0.81 (p = 0.037) and RSE  = 0.59 CI. These models (*CI*  = 5.84×10−^11^× *Q* ^4^.^304^; and *CI* = −8.07+0.0364 *Q*) predicted the 2011 bloom within 35%: a CI of 19.0 (exponential) and 9.15 (linear) vs 14.2 observed in 2011.

## Discussion

Spring flow and loads explain the severity of cyanobacterial blooms in Lake Erie. The lag of up to two months between the spring load and peak biomass allows sufficient time for recycling of TP and dispersion in the basin to support growth under optimal temperature (Figure 6) and light conditions. The results here show that the bloom severity can be modeled solely with nutrient influx, as argued by others [34]. This leads to a set of models that may permit prediction of these blooms:

CI from Q exponential:


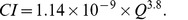
(3)

CI from TP_June_ (without 2004 and 2011)


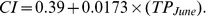
(4)

The exponential model with Q captures the reasonable non-linear response at low loads. But why is Q the more effective predictor? SRP loads may promote spring blooms near the river mouth [35]. TP, which is not immediately usable and may settle, can be recycled to usable forms by bacteria and Dreissenid mussels [8]–[9]. Subsequent spring discharge would disperse this bioavailable P across the western basin. The Maumee River may also be a source of cyanobacteria [35], although this is debated [36].

The model was applied to data going back to 1995 (Figure 8), the first of the recent blooms [37]. The model correctly hindcasts negligible blooms from 1999–2001 and indicates a bloom of 1.6 CI in 1998 (size between the 2004 and 2003 blooms). Before 1998 the model fails; it hindcasts a 2003-size bloom in both 1996 and 1997 when none were reported, and no bloom for 1995, when one occurred [38]. This change in predictive skill follows the colonization of the lake by Dreissenid mussels, which fundamentally altered the way carbon and nutrients are recycled and packaged [3], [38]. We hypothesize that the impact of the Dreissenid mussels on cyanobacteria took several years, beginning before 1995 and changing to a new ecological state by 1998 that increased the importance of the Maumee phosphorus loads on these blooms.

With the relatively short 10-year period of study, there are other questions of environmental variability. In the winter of 2011–2012, for example, Lake Erie did not freeze over. Could this influence the forecasts? While infrequent, lack of ice is not unusual; Lake Erie also had minimal ice cover in 1998, 2002, and 2006 [39]. Of these years, 1998 had a reported bloom, while 2002 and 2006 had minor blooms. Ice cover does not point in either direction at this time. Of note, when the lake is ice covered, it can have dense winter diatom blooms under the ice [40]–[41]. Without ice cover, there can be greater sediment resuspension, potentially further reducing light availability until spring.

### Seasonal Forecast

A seasonal forecast of the bloom severity may be possible from the models presented here (equations 3 and 4). This forecast could be made in early July, nearly two months before the bloom reaches its peak. Q is available provisionally from the USGS within a day, and TP is available from NCWQR within a week. The exponential model with Q for March to June (equation 3) would provide the primary forecast. TP for June (equation 4) can provide a comparative reference, except when the June load is much larger than that for March to May (Table 1). If the June TP load is much larger than the March-May load, namely the ratio of TP_June_/TP_March-May_ >0.2 (henceforth TP ratio), which occurred in 2004, 2001, and 2000, the June TP model appears to be inappropriate (Figure 8). (A relatively low TP in June may matter only when March-May is extremely high as in 2011).

As opposed to using only the exponential Q model (equation 3); blending the Q model with the TP_June_ model can produce lower RMS error, and therefore a more robust model. For any year when the TP ratio > = 0.2, the Q model may be used. For years that have a TP ratio <0.2, the average of the two models may be more appropriate. Using the Q model alone on the 10 years had a Root Mean Square (RMS) error of 0.55 CI. By using the blended model (except for 2011, when TP_June_ was severely low relative to the extreme March to May load), the RMS error decreases to 0.37 CI (an uncertainty in nominal area of 105 km^2^ vs. 165 km^2^ for the Q only model).

### Conclusions

Seasonal forecasts of significant ecological hazards are not common. Three noteworthy forecasts are the annual “red tide” in the Gulf of Maine, and the summer hypoxia in both the Chesapeake Bay and the Gulf of Mexico [42]–[43]. The results here indicate that spring nutrient load explains the cyanobacteria bloom in Lake Erie such that Q and TP allow for a new seasonal forecast. The importance of spring discharge and phosphorus loads may provide insight into strategies for targeted reductions of these loads [6], rather than pursuing reductions of the annual load.

We propose to issue a forecast at the start of the season (shortly after July 1). The forecast will identify the intensity of the cyanobacteria bloom and the equivalent area covered by bloom conditions. The peak intensity and nominal area of the bloom will be predicted in both quantitative and qualitative forms. Qualitative categories could be minimal, moderate, and severe. Our ability to predict bloom extent is based on the models presented here, and is not dependent on additional ocean color data. Skill assessment of the model and adding additional data points, however, are important and will be dependent on the acquisition of new satellite imagery. With the loss of MERIS data in April 2012, validation will be more difficult until 2014 when the European Space Agency plans on launching a successor to MERIS, called the Ocean and Land Colour Instrument (OLCI), on the Sentinel-3 satellite. This new sensor would continue the capability demonstrated by MERIS into the future for further model development and skill assessment.
